# Craniofacial Cephalometric Morphology in Caucasian Adult Patients with Cleft Palate Only (CPO)

**DOI:** 10.3390/diagnostics13122058

**Published:** 2023-06-14

**Authors:** Alicja Zawiślak, Barbara Wędrychowska-Szulc, Katarzyna Grocholewicz, Joanna Janiszewska-Olszowska

**Affiliations:** 1 Department of Orthopaedics and Orthodontics, Institute of Mother and Child, ul. Kasprzaka 17a, 01-211 Warszawa, Poland; alizawislak@imid.med.pl; 2 Department of Interdisciplinary Dentistry, Pomeranian Medical University, al. Powstańców Wlkp 72, 70-111 Szczecin, Poland; katarzyna.grocholewicz@pum.edu.pl; 3 Private Praxis Vita-Med 2, Ledochowskiego 16/U3A, 71-004 Szczecin, Poland; bwedrychowska@gmail.com

**Keywords:** cleft palate, cleft palate only, isolated cleft palate, cephalometry, cephalometric analysis, craniofacial morphology

## Abstract

Orofacial clefts are common birth defects that affect the morphology of the skull. Cleft palate only (CPO) has a different etiology than other types of clefts, and craniofacial morphology in CPO differs from that of UCLP and BCLP. The long-term effect of the cleft and its surgery is visible after growth cessation. However, few studies exist describing cephalometric craniofacial morphology in adults with CPO. The aim of the present study was to describe the cephalometric craniofacial morphology of adult patients with CPO compared to healthy patients. The study included analysis of cephalometric lateral headfilms of 28 adults with CPO and 28 healthy subjects. It was found that the angles of SNA, ANB, 1-:NB angle (°) and Wits appraisal were significantly smaller in CPO, whereas NL-NSL (°), 1+:NA angle (°) and 1+:NA (mm) had significantly higher values in CPO compared to the control group. It has been concluded that CPO in adult patients is characterized by a sagittal jaw discrepancy due to maxillary deficiency, with a tendency for compensatory inclination of the upper and lower incisors.

## 1. Introduction

Craniofacial cleft anomalies, also known as orofacial clefts, are a group of congenital defects that affect the facial and oral structures of newborns. They represent the second most prevalent type of congenital defect in children worldwide. The incidence of craniofacial cleft anomalies varies depending on several factors, including geographic location, ethnic background and socio-economic status. The incidence ranges from 1/300 to 1/2500 live births. In Europe, the incidence of orofacial clefts (ORFs) is estimated to be around 1 in 1000 live births. This prevalence rate highlights the significance of craniofacial cleft anomalies as a health challenge in society. The management of these defects requires the implementation of intricate and multidisciplinary surgical, orthodontic, dental, phoniatric and psychological interventions [1].

Craniofacial cleft anomalies can affect both the skeletal framework and the soft tissues of the face and oral cavity at macroscopic and histological levels. The range of affected structures in cleft defects depends on several factors, including the developmental timing of the anomaly during embryogenesis and factors influencing the embryo [2].

The primary morphological anomaly observed in craniofacial cleft defects is the lack of tissue continuity within the lip, alveolar process, hard and soft palate or any combination of these structures. In certain types of clefts, such as submucous cleft lip or bifid uvula, the anatomical disruptions may be minor. In other cases, the anomalies may be more severe, with the most significant morphological and functional disturbances observed in complete unilateral and bilateral clefts of the primary and secondary palate.

Craniofacial cleft anomalies require the implementation of a range of intricate and multidisciplinary interventions, highlighting the importance of collaboration between different medical disciplines to ensure the best possible outcomes for affected individuals [3,4].

The term “cleft palate” is used in the literature for different types of clefts [5], whereas the term “isolated cleft palate” is used for clefts of the palate only [6,7] and for all types of non-syndromic clefts [8,9]. This makes it difficult to search for studies and communicate between clinicians. Thus, the authors of the present study decided to use the term “cleft palate only” (CPO) throughout the entire paper.

Cephalometric studies indicate that craniofacial morphology in patients with CPO differs from those with UCLP and BCLP [9,10]. The cephalometric craniofacial morphology in adult patients reflects both the severity of the cleft and long-term results of treatment.

Few studies exist describing the cephalometric craniofacial morphology of patients with CPO [6,10,10,11,12,13,14,15,16]. Moreover, they are difficult to find in scientific databases due to the use of different terminology. Some data are also available in studies comparing patients with different types of clefts. A study dealing with infants could be found [17], which does not refer to orthodontic or orthognathic treatment. On the contrary, few studies refer to adult CPO patients.

The aim of the present study was to describe the cephalometric craniofacial morphology of adult patients with CPO compared to generally healthy orthodontic patients: to verify the presence of maxillary and mandibular deficiency, to describe the degree of sagittal jaw discrepancy and to define other cephalometric characteristics of CPO patients.

## 2. Materials and Methods

The study sample comprised 28 lateral cephalograms (selected from all cephalograms taken in a cleft center in the years 2003–2020) of Polish patients (13 female and 15 male) with CPO aged from 18 to 23 (mean age 20.23 years). The inclusion criteria for the study group comprised CPO, age 18 or older at the moment of performing cephalometric radiograph and a good quality of lateral headfilm. The matching control group (*n* = 28) was recruited from consecutive patients referred for orthodontic treatment aged from 18 to 28 (16 female and 12 male, mean age 24.35 years). The inclusion criteria for both groups were: Polish origin, Caucasian, age 18 years or older, and sufficient quality of the cephalogram for the identification of all cephalometric landmarks; moreover, for the study group, cleft palate only, and for the control group, no craniofacial deformity.

All patients from the study group were operated on (before the end of the first year of life) using a modified Langenbeck method [18]. All cephalograms were made by the use of digital X-ray device (Cranex Tom, Soredex, Finland). They were analyzed in November and December 2021.

**Figure 1:**
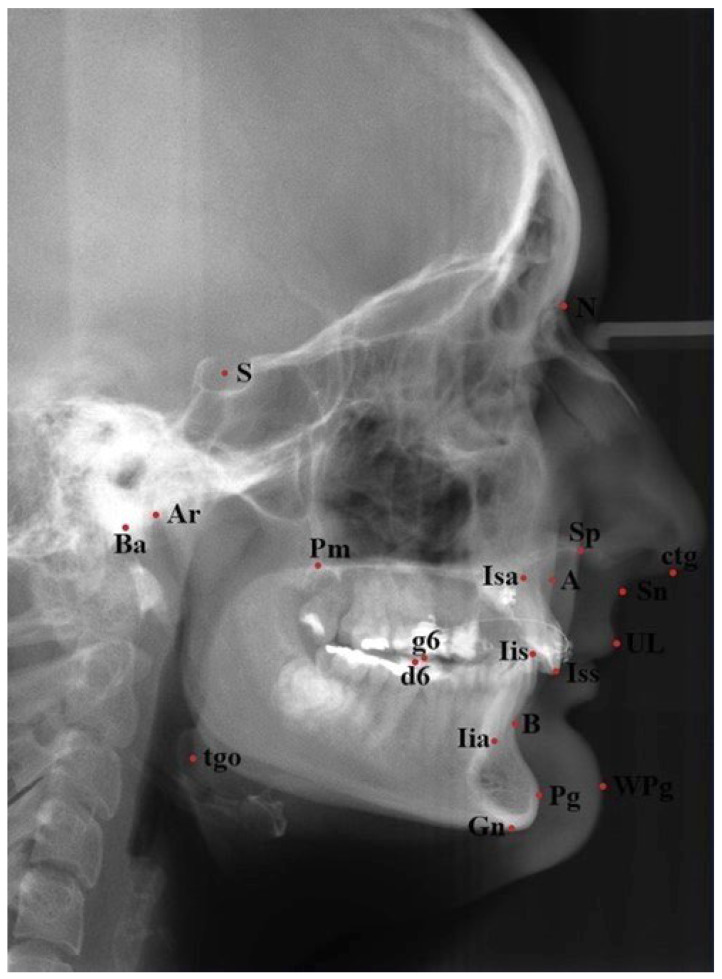
Cephalometric landmarks used in this study.

The digital X-rays received were analyzed in specialized cephalometric software Ortodoncja 7.0 (Orto-Bajt, Wroclaw, Poland) according to Segner and Hasund [19]. Cephalometric landmarks used for the purpose of this study are presented in Figure 1. Cephalometric variables used are presented in Table 1.

Every analysis was performed by the first and the senior authors, and inter-examiner reliability was verified using ICC (intraclass correlation coefficient). A mean value between the two measurements was used for further comparisons and analysis of correlations. Two weeks later, twenty-one randomly selected cephalograms were reanalyzed by the same investigators and inter- and intraexaminer reliability was assessed with ICC (intraclass correlation coefficient). The interpretation of the ICC values according to Cicchetti et al. (1994) [20] was adopted: ICC above 0.75 means excellent accordance, ICC 0.6–0.75—good, ICC 0.4–0.6 mean, and ICC below 0.4—a weak accordance between the measurements.

Correlations of cephalometric variables with age were analyzed. Relationship between two quantitative variables was assessed with Spearman’s correlation coefficient. Comparisons were made between the study and control groups using Chi-squared test (with Yates’ correction for 2 × 2 tables) to compare qualitative variables among groups. In case of low values in contingency tables, Fisher’s exact test was used instead. Mann–Whitney test was used to compare quantitative variables between two groups. Significance level for all statistical tests was set to 0.05. R 4.0.5 was used for computations.

## 3. Results

Excellent interexaminer reliability for all the measurements was demonstrated according to the methodology employed by Cicchetti et al. (1994) [20]. The intraexaminer reliability of the first author was excellent for all measurements and, of the other one, for most measurements (good for NSBa, NL-NSL, ML-NL) according to Cicchetti et al. [20].

No patients from the study or control group had orthognathic surgery. The distribution of cephalometric variables in the study and control groups is presented in Table 2.

Values *p* < 0.05 indicating statistically significant differences were stated for SNA, ANB, 1-:NB angle and Wits, which were significantly lower in the study group. NL-NSL, 1+:NA angle and 1+:NA were significantly higher in the study group.

**Figure 2:**
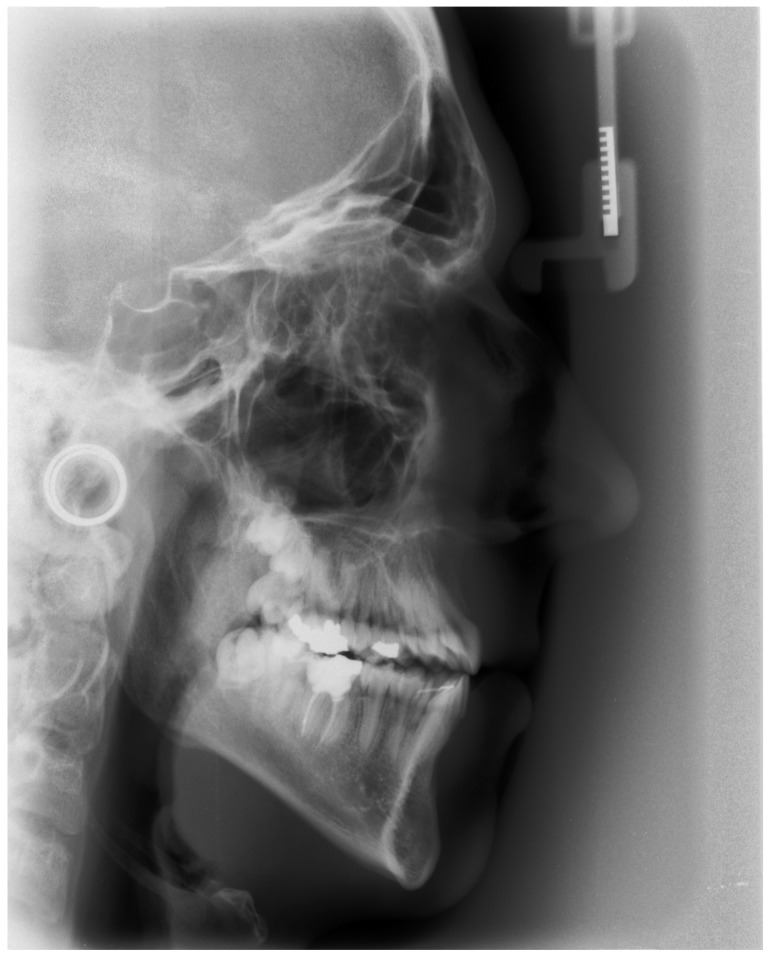
Cephalometric radiograph of a patient from the study group.

An example of a cephalometric radiograph of a patient from the study group presenting characteristic features of the craniofacial morphology is presented in Figure 2. A protrusion of the upper incisors is visible as a compensation for the sagittal skeletal discrepancy. The lower incisors are steep.

## 4. Discussion

Numerous methods of cephalometric analysis have been described and are used worldwide. The method by Segner and Hasund [19] used for cephalometric analysis in the present study comprises numerous angular and linear measurements of the soft and hard tissues. It is considered to be an individualized method, taking into account overall facial morphology and “facial type” when defining the individual patient’s horizontal and vertical discrepancy. The norms were established based on a study of the German population and, thus, could be reliably applied to the study and control groups of the present study.

ICC for repeated cephalometric landmark identification on 2D lateral cephalometric radiographs by experienced clinicians in the literature is 0.8 [21,22]. No studies have been found referring to the repeatability of landmark identification in patients with cleft palate. The first, second and the senior authors are experienced in cephalometric analysis of patients with clefts; thus, the relatively worse intraexaminer reliability (comparing to literature) for values comprising maxillary base probably results from the absence of bony palatal structures and, thus, difficult identification of some landmarks.

The ANB angle in the group of patients with CPO of a mean −1.97 is below normal reference values of cephalometric analysis. Compared to the control group, ANB is significantly lower, reflecting a sagittal discrepancy resulting from the cleft. The age range of the study group is 18–23 years, enabling a slight potential for worsening sagittal jaw relation, as the ANB angle tends to decrease with age, but it does not prognose a severe discrepancy. According to Segner and Hasund [19], the mean reduction in the ANB angle between the ages 6 and 16 is 2.5° and, still, a minimal mean change is noted between ages 18 and 19 in men. However, these values refer to healthy patients without craniofacial abnormalities who, thus, may be different in severe malocclusion and clefts. The Wits appraisal was negative in the CPO group, as well. According to the literature, this measurement describes true sagittal discrepancy, independent of mandibular rotation, and does not tend to worsen with age [23]. In the present study, the negative Wits appraisal in the CPO group confirms a skeletal sagittal discrepancy between the maxilla and the mandible.

Maxillary morphology is described by the SNA value in the majority of methods used by clinicians for cephalometric analysis, despite the fact that it changes with protrusion or retrusion of the upper incisors, since the A point is positioned on the alveolar process. Referring to mean SNA in CPO, slightly higher values were reported by Ye Z. et al. (2013) [24] in unoperated CPO patients older than 16 y.o., where the SNA in the study (CPO) group equaled 79.3 ± 4.39. This confirms that palatal surgery is a factor restricting maxillary sagittal growth. In the control (non-cleft) group, SNA was 80.27 ± 3.62, which is consistent with the present study (80.97 ± 3.54). The study by Xu Y. et al. (2014) [25] found similar SNA angles in adult unoperated CPO patients, 79.4 ± 4.7, whereas, in the control (non-cleft) group, it averaged 81.4 ± 3.9. The differences between the groups were statistically insignificant. In the studies by Heliövaara et al. (2009) [13] on 7-year-old boys with operated sCPO as well as by Heliövaara et al. (2003) [8] on 6-year-old girls with operated CPO, mean SNA was reduced, indicating a maxillary deficiency, consistent with the present study.

Significantly lower SNA values in the study compared to the control group confirm a maxillary deficiency. Its severity presents a full manifestation at the cessation of growth. Thus, the sagittal jaw discrepancy is purely caused by a maxillary deficiency. Contrary results referring to differences in SNA between CPO and non-cleft patients were reported by Ye Z. et al. (2013) as well as by Xu Y. et al. (2014) [24,25] in unoperated CPO patients. This confirms that palatal surgery and the resulting tissue scarring significantly contribute to the severity of maxillary deficiency in CPO.

A recent study by Tsuji et al. [6], describing craniofacial cephalometric morphology in CPO on 5-year-old girls (*n* = 36), revealed a bimaxillary retrusion (mean values: SNA = 80.1, SNB = 76, ANB = 4.1). A lack of significant differences referring to SNB and SNPg in the present study indicates that any mandibular deficiency in the study group (if present at earlier ages) is overcome during growth.

Maxillary inclination to cranial base in the present study is statistically significantly higher in CPO compared to the control group, reflecting a downwards maxillary rotation. However, the lower anterior face height is normal, as described by the Index value (proportion in percentage between the middle and lower face height). In contrast to the present study, Tsuji et al. [6] found an increased gonial angle in 5-year-old girls with CPO. In contrast to CPO, a reduced anterior maxillary height is a common finding in operated patients with UCLP [15].

The dentoalveolar compensation is expressed in incisor inclinations, e.g., in CPO patients, the upper incisors are more proclined and the lower are retroclined compared to the control group. Similar findings were reported by Smahel et al. (1999) [26]. It should be underlined that protrusion of the upper incisors in CPO patients in the present study partially compensates the skeletal discrepancy and, by causing a more anterior position of the A point, masks the true sagittal maxillary deficiency, influencing the values of the SNA and ANB angles.

Ye et al. 2013 [24] described the craniofacial cephalometric morphology of unoperated CPO patients compared to non-cleft individuals. No studies have been published comparing the craniofacial cephalometric morphology of operated versus non-operated CPO patients. A recent meta-analysis by Janiszewska-Olszowska et al. (2022) [27], based on studies comparing unoperated and operated CPO patients to non-cleft individuals, indicated that maxillary deficiency in CPO is both due to the cleft itself and to the palatal surgery. Differences in craniofacial morphology have been confirmed between patients of different cleft severity. It was confirmed in the meta-analysis cited above that submucous CPO is associated with a lesser degree of maxillary deficiency compared to visible CPO. Thus, it could be expected that differences exist between patients with Veau I vs. Veau II clefts.

Moreover, in the meta-analysis by Janiszewska-Olszowska et al. (2022), ethnic differences referring to craniofacial morphology were confirmed. This finding indicates that the results of the present study probably apply to European patients, but the craniofacial morphology may differ for other populations.

Concerning the soft tissue profile, neither H angle (referring the soft tissues of upper lip and chin to the skeletal structures represented by the Nasion and B points) nor the nasolabial angle (NLA) are significantly different in CPO compared to the control group. Thus, unlike in UCLP and BCLP, facial morphology and appearance are not severely altered compared to non-cleft patients. As far as the literature is concerned, one study (da Silva Filho et al., 2007) [28] was found describing subjective profile assessment (on facial photographs) of a mixed sample of patients with UCLP and CPO (*n* = 85), referring to NLA, facial pattern and zygomatic projection. However, the study group consisted of patients with different cleft types, and the conclusions refer to no negative effect of palatoplasty on the profile. Thus, the results cannot be referred to for the present study.

Nevertheless, it would be very interesting to investigate a different severity of velopharyngeal disfunction in CPO, not always indicating a need for a surgical intervention.

Following a search in scientific databases, seven studies referring to cephalometric craniofacial morphology in adult CPO patients could be found in the literature, based on various measurements (Table 3).

The studies cited above were published in the years 1992–2017 and included between 14 and 189 patients with different types of orofacial clefts. It was difficult to find relevant papers due to the use of different terminology describing cleft types in the studies. Cephalometric analysis in the studies tabularized above was made using various landmarks, measurements and various methods of cephalometric analysis; thus, it was difficult to compare the results obtained from different studies. Moreover, the previous studies have different designs, and the aims differ between the investigations. The papers cited refer to: comparison of craniofacial morphology in patients with different types of orofacial clefts [10,26], comparison of craniofacial cephalometric morphology in patients with CPO to those with sCPO (submucous cleft palate only) or even to the benefit of 3D cephalometrics [10]. Moreover, it would be interesting to analyze the physical characteristics of the patients (height, weight, build) included in the groups, to find out if these features could affect craniofacial morphology.

Possible limitations of the present study may include its retrospective design. It is noteworthy that no standard protocols of orthodontic treatment exist for CPO patients in terms of timing, techniques or cooperation with other specialties. Since ethnic differences exist, more studies on various ethnic groups of CPO patients could be performed in the future. It should also be underlined that the results of the present study may not refer to all populations. It is important that terminology is unified and study groups are homogenous (patients with different cleft types should not be included). As altered craniofacial morphology results both from the cleft and from surgery, more homogenous studies on CPO patients are needed to learn whether and how orthodontic or orthognathic correction could possibly avoid future craniofacial abnormalities. Expanding the existing knowledge could allow for establishing reliable evidence-based clinical recommendations.

It is necessary to underline that the field of orthodontics has witnessed significant advancements in technology in recent years. The integration of implementing new technologies and methods into orthodontics has led to significant changes in craniofacial imaging, with traditional methods giving way to more advanced techniques. These advancements have led to significant changes in craniofacial imaging, with traditional methods, such as cephalometric and intraoral radiographs, as well as manual cephalometric measurements, giving way to modern techniques, including digital imaging and intraoral scanners. One of the most significant changes in the field of orthodontics is the rise of software-assisted cephalometric tracings. This newer technology has allowed for more precise and comprehensive analysis of craniofacial structures, leading to more accurate diagnoses and better treatment planning [31,32].

Particularly, the integration of AI into various fields has led to significant advancements in technology. AI in orthodontics is an exciting development, as it offers the potential to revolutionize the field, providing a more comprehensive view of a patient’s anatomy. In the field of orthodontics, AI has become increasingly popular as a tool for identifying cephalometric landmarks and analyzing cephalometric radiographs, introducing 3D image analysis for orthodontic diagnosis and treatment planning. Deep learning algorithms have been developed to automatically mark cephalometric points in 3D scans, and attempts have been made to use them in patients with cleft lip and palate. This technology seems to be very promising referring to analysis of craniofacial morphology in patients with orofacial clefts based on 3D scans. However, it is suggested that the training sets should be expanded to improve the accuracy [31,32,33]. While landmarks on the mandible can vary significantly between subjects, those on the cranium are relatively stable. Therefore, cranial landmarks are better suited for effective estimation and identifying common patterns across the training dataset [34]. Moreover, it should be underlined that in orofacial clefts, the identification of anatomical abnormalities is crucial [26]; however, as observed in the present study, landmark identification is more difficult due to altered skeletal morphology.

It is very important to note that all software used for cephalometric analysis of 3D scans currently uses 2D analysis from 3D scans. A true 3D analysis should use volumes and surface areas rather than linear and angular measurements. This would require creating completely new 3D cephalometric norms from CBCT scans of healthy patients at different ages. The norms should then gain wide acceptance from the community of specialists in orthodontics and craniofacial surgery. However, according to the ALARA rule, radiography can only be performed if medical indications exist and the potential diagnostic benefits overweigh the harm of radiation. It seems unlikely that a shift from 2D to true 3D cephalometric analysis could be observed in the near future. It could be expected that the use of artificial intelligence in the future should allow for accurate and comprehensive true 3D analysis of the craniofacial morphology. Possible true 3D automatic analysis via algorithms of the artificial intelligence would require rather automatic segmentation of craniofacial structures than automatic landmark identification. As these technologies continue to develop, it is likely that we will see even more advancements in the field of craniofacial orthodontics in the coming years, with the development of more advanced algorithms and tools for analysis of craniofacial morphology [32,34,35]. This could enable more detailed and comprehensive analyses of patients with orofacial clefts based on 3D scans, allowing for better understanding and treatment of these complex conditions [35].

## 5. Conclusions

CPO in adult patients is characterized by a sagittal jaw discrepancy due to maxillary deficiency, with a tendency for compensatory inclination of the upper and lower incisors. Unifying the nomenclature and performing more studies on CPO patients could expand the existing knowledge and, thus, allow for establishing evidence-based clinical recommendations.

## Figures and Tables

**Table 1:** Cephalometric variables used (according to Segner and Hasund, 1998) [19].

Abbreviation	Mean Value	Interpretation	Special Significance in Cleft Palate (Only)
SNA	82	Sagittal maxillary position referring to cranial base.	Negative—indicates sagittal maxillary deficiency.
SNB	80	Sagittal position of the mandibular alveolar part referring to cranial base.	Reduced in mandibular deficiency.
ANB	2	Sagittal relation between the maxilla and mandible.	Negative in sagittal maxillary deficiency referring to mandible, reduces with age due to normal growth.
SNPg	82	Sagittal position of the chin referring to cranial base.	Reduced in mandibular deficiency.
NL-NSL	8	Vertical maxillary inclination relative to cranial base.	Reduced in vertical maxillary deficiency.
ML-NSL	28	Vertical mandibular inclination relative to cranial base.	Increased in posterior rotation of the mandible.
ML-NL	20	Vertical jaw relation.	Increased in posterior rotation of the mandible and in vertical maxillary deficiency.
NS-Ba	130	Inclination of the clivus to cranial base.	_
Gn-tgo-Ar	122	Gonial angle.	Increased in severe mandibular deficiency with posterior rotation
H	9.2	Angle between the line upper lip—soft-tissue chin relative to line NB—inclination of the soft tissue profile.	Reduced in upper lip retrusion associated by maxillary deficiency, reduces with normal growth.
1+:1-	133	Angle between the long axes of upper and lower central incisors.	_
1+:NA	21	Upper incisor inclination to NA line.	Increased with protrusion of upper incisors (compensatory to sagittal maxillary deficiency).
1+:NB	24	Lower incisor inclination to NB line.	Reduced with retrusion of the lower incisors (compensatory to sagittal jaw discrepancy)
Nasolabial angle	110	Angle between nasal base and upper lip.	Increased in sagittal maxillary deficiency.
Index	80	Proportion between the upper and lower face height (in percentage).	Reduced in vertical maxillary and midface deficiency, reduced in posterior mandibular rotation.
Pg:NB (mm)	2.3	Distance between the point Pg and NB line. Describes chin prominence.	Reduced in mandibular Deficiency
1+:NA mm	4	Distance between the incisal edge of the upper central incisor and NA line.	Increased in protrusion of upper incisors (compensatory to sagittal maxillary deficiency).
1-:NB mm	3.8	Distance between the incisal edge of the lower central incisor and NB line.	Reduced in retrusion of lowers incisor (compensatory to sagittal jaw discrepancy).
Wits (mm)	0	Distance between perpendicular projections of points A and B on the occlusal plane.	Negative value in maxillary deficiency.

SNA—Sella-Nasion-Point A angle; ANB—Point A-Nasion-Piont B angle; NSBa—Nasion-Sella_Basion angle; SNB—Sella-Nasion-Point B angle; NLA—nasolabial angle; WITS—WITS appraisal.

**Table 2:** Distribution of cephalometric values for patients older than 18 years in the study group (*n* = 28), control group (*n* = 28) and significance of differences between the groups.

Variable		Group		*p*
		Control Group (*n* = 28)	Study Group (*n* = 28)	
SNA (°)	mean ± SD	80.97 ± 3.54	77.18 ± 4.36	*p* = 0.001 *
	Median	80.7	77.2	
	Quartiles	78.7–83.62	74.8–79.2	
SNB (°)	mean ± SD	78.17 ± 3.87	79.15 ± 5.43	*p* = 0.857
	Median	78.15	77.95	
	Quartiles	76.42–80.7	76.77–80.65	
ANB (°)	mean ± SD	2.92 ± 2.71	−1.97 ± 4.8	*p* < 0.001 *
	Median	2.95	−1.75	
	Quartiles	0.98–4.68	−4.05–1.35	
SNPg (°)	mean ± SD	79.43 ± 3.9	80.51 ± 5.67	*p* = 0.928
	Median	80.3	79.9	
	Quartiles	77.3–82.15	78.22–82.32	
NSBa (°)	mean ± SD	130.08 ± 5.91	130.33 ± 6.14	*p* = 0.902
	Median	130.6	129.95	
	Quartiles	126.33–133.27	127.17–132.92	
GntgoAr (°)	mean ± SD	123.08 ± 7.42	127.45 ± 9.1	*p* = 0.088
	Median	122.95	126.1	
	Quartiles	118.83–125.75	119.8–134.62	
NL-NSL (°)	mean ± SD	7.45 ± 3.62	11.53 ± 5.43	*p* = 0.004 *
	Median	7.5	11.95	
	Quartiles	4.53–9.32	7.18–15.45	
ML-NSL (°)	mean ± SD	30.7 ± 7.16	33.83 ± 8.63	*p* = 0.142
	Median	29.7	32.7	
	Quartiles	26.6–34.95	29.82–37.42	
ML-NL (°)	mean ± SD	23.23 ± 7.22	22.35 ± 9.24	*p* = 0.941
	Median	22.25	21.6	
	Quartiles	18.55–28.95	15.52–28.58	
H	mean ± SD	9.82 ± 5.37	7.78 ± 5.42	*p* = 0.068
	Median	9.15	5.95	
	Quartiles	6.57–13.35	3.8–11.1	
+:1- angle (°)	mean ± SD	132.66 ± 14.08	135.59 ± 13.17	*p* = 0.413
	Median	131	131.65	
	Quartiles	122.88–139	126.35–143.52	
1+:NA angle (°)	mean ± SD	20.09 ± 10.41	26.87 ± 9.97	*p* = 0.035 *
	Median	21.25	27.2	
	Quartiles	17.5–26.52	20.15–34.55	
1-:NB angle (°)	mean ± SD	24.42 ± 7.6	19.23 ± 8.22	*p* = 0.025 *
	Median	25.95	19.95	
	Quartiles	19.68–30.05	16.7–24.88	
Nasolabial angle (°)	mean ± SD	109.83 ± 11.12	102.64 ± 18.62	*p* = 0.152
	Median	110.6	105.9	
	Quartiles	104.77–117.75	92.23–116	
Pg:NB [mm]	mean ± SD	1.69 ± 1.58	1.9 ± 1.52	*p* = 0.566
	Median	1.35	1.8	
	Quartiles	0.5–2.68	0.9–2.47	
1+:NA [mm]	mean ± SD	2.48 ± 3.09	4 ± 2.39	*p* = 0.018 *
	Median	2.05	3.3	
	Quartiles	1.03–3.45	2.28–6.08	
1-:NB [mm]	mean ± SD	2.71 ± 2.23	2.26 ± 2.39	*p* = 0.928
	Median	2.1	2.6	
	Quartiles	1.1–3.2	0.92–3.55	
Index	mean ± SD	78.46 ± 8.49	77.78 ± 12.02	*p* = 0.441
	Median	78.8	74.3	
	Quartiles	72.45–83.75	71.05–82.72	
Wits [mm]	mean ± SD	0.39 ± 2.76	−4.02 ± 4.63	*p* < 0.001 *
	Median	0.6	−3.1	
	Quartiles	−1.08–2.22	−6.17–−1.15	

*p*—Mann–Whitney test; * statistically significant (*p* < 0.05).

**Table 3:** Studies on cephalometric analysis on adult CPO patients (from most recent to oldest).

Author	Year	Characterictics of Subjects					Cephalometric Measurements
		Number of Subjects with CPO	Origin	Gender	Mean Age (Age Range)	Characteristics of Malformation	
Cao C et al. [29]	2017	40	Chinese	F/M	CPO (25.43 ± 7.18, sCPO 24.32 ± 6.22)	CPO, sCPO	S-N, S-Ba/S-N, N-Ba/S-N, ANS-Me/S-N, Ba-PMP/S-N, Ba-ANS/S-N, A-PMP/S-N, PMP-ANS/S-N, ANS-N/S-N, R-PMP/S-N, Ar-Go/S-N, Pog-Go/S-N, N-Me/S-N, < Ba-N-ANS, <S-N-ANS, <S-N-A, <S-N-B, <S-N-Pog, <N-S-PMP, <Ar-Go-Gn, <A-N-B
Antonarakis G.S. et al. [14]	2015	189	Caucasian, Asian, African	F/M	Minimum age of 15 years	CPO	maxillary length, maxillary protrusion, maxillary height, maxillary inclination
Xu Y. et al. [25]	2014	30	Chinese	F/M	Over 18	CPO	S-N, S-Ba, N-Ba, NSBa, Pmp-Ba, Pmp-S, ANS-Pmp, N-ANS, Pmp-NSL, SNA, Lo-Lo’, Mo-Mo’, Apt-Apt’, Mx-Mx’, Zyg-Zyg’, Gn-Go, Cd-Go, Gn-Cd, Ii-Pgn, SNB, SnPg, SN/GoPgn, ANSPmp/Go-PGn, Cd-Cd’, Go-Go’, ANB
Ye Z et al. 2013 [24]	2013	37	Chinese	F/M	22.19 ± 6.57	CPO	N-S/mm, N-Ba/mm, S-Ba/mm, Ba-S-N/°, ANS-Me, N-ANS, N-Me, S-Ptm, Pog-Go, Ar-Go, R-PMP, Ba-PMP, PMP-ANS, PMP-A, ∠SNA, ∠SNB, ∠ANB, Ba-N-ANS, Ba-N-A, S-N-ANS, S-N-Pog, SN-PP, MP-SN, Ar -Go-Me, N-ANS/N-Me, R-PMP/N-ANS
Diah E et al. [10]	2007	92	Indian	F/M	21.6 (range 16–47 years)	UCL, UCLP, BCLP, CPO	SNA
Smahel Z et al. [26]	1999	34 complete CPO + 34 incomplete CPO + 17 sCPO	Czech	M	(20–40)	UCL, UCLP, BCLP, CPO	SNA, SNB, ANB, Spp-Spa, S-Go%N-Me, Is-NPo, overjet, Ls-EL
Yoshida H et al. [30]	1992	14	Chinese	F/M	13–28	CPO	SNA, ANS-Ptm, N-ANS, SNB, mandibular plane angle, facial angle, ANB, U1-SN

CPO—cleft palate only; sCPO—submucosus cleft palate only; UCL—unilateral cleft lip; UCLP—unilateral cleft lip and palate.

## Data Availability

All data are available from corresponding author on reasonable request.
